# Fetal Gene Reactivation in Pulmonary Arterial Hypertension: GOOD, BAD, or BOTH?

**DOI:** 10.3390/cells10061473

**Published:** 2021-06-11

**Authors:** Sarah-Eve Lemay, Charifa Awada, Tsukasa Shimauchi, Wen-Hui Wu, Sébastien Bonnet, Steeve Provencher, Olivier Boucherat

**Affiliations:** 1 Pulmonary Hypertension Research Group, Centre de Recherche de l’Institut Universitaire de Cardiologie et de Pneumologie de Québec, Québec, QC G1V 4G5, Canada; sarah-eve.lemay@criucpq.ulaval.ca (S.-E.L.); charifa.awada.1@ulaval.ca (C.A.); tsukasa.shimauchi.1@ulaval.ca (T.S.); wenhui5621006@126.com (W.-H.W.); Sebastien.Bonnet@criucpq.ulaval.ca (S.B.); Steeve.Provencher@criucpq.ulaval.ca (S.P.); 2 Department of Cardio-Pulmonary Circulation, Shanghai Pulmonary Hospital, Tongji University School of Medicine, Shanghai 200433, China

**Keywords:** vascular remodeling, cardiac hypertrophy, right ventricular failure, development

## Abstract

Pulmonary arterial hypertension is a debilitating chronic disorder marked by the progressive obliteration of the pre-capillary arterioles. This imposes a pressure overload on the right ventricle (RV) pushing the latter to undergo structural and mechanical adaptations that inexorably culminate in RV failure and death. Thanks to the advances in molecular biology, it has been proposed that some aspects of the RV and pulmonary vascular remodeling processes are orchestrated by a subversion of developmental regulatory mechanisms with an upregulation of a suite of genes responsible for the embryo’s early growth and normally repressed in adults. In this review, we present relevant background regarding the close relationship between overactivation of fetal genes and cardiopulmonary remodeling, exploring whether the reawakening of developmental factors plays a causative role or constitutes a protective mechanism in the setting of PAH.

## 1. Introduction

**Figure 1:**
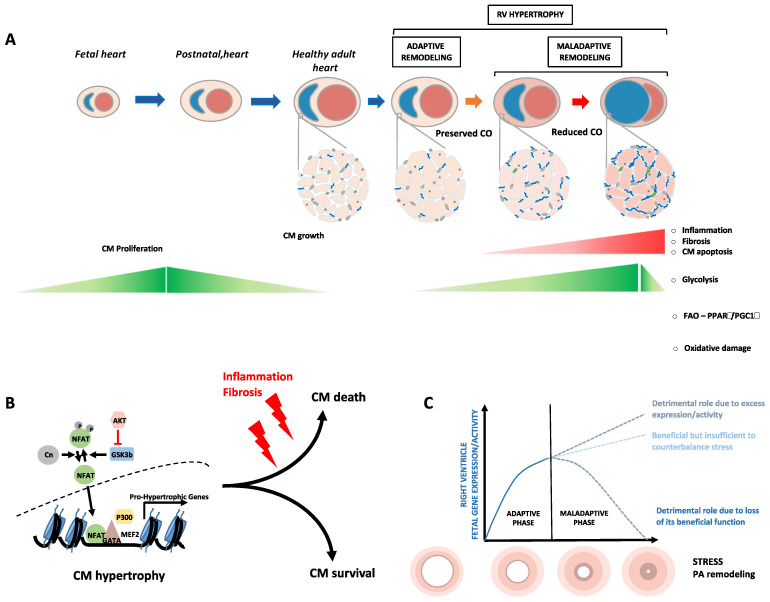
**Relationships between cardiac development and RV remodeling.** (**A**) During lung morphogenesis, proliferating cardiomyocytes (CMs) are primarily reliant on glycolysis for energy production. At least in part driven by the hypoxic to normoxic transition at birth, a metabolic switch from glycolysis to fatty acid oxidation occurs contributing to a gradual loss of CM proliferative capacity and their enhanced maturation. In response to stress, such as increase pulmonary artery pressure, cardiomyocytes experience cellular and molecular changes to mitigate wall stress including a hypertrophic growth with a reactivation of a fetal metabolic profile (preference of carbohydrates over fatty acids as substrates for energy) and re-expression of contractile proteins normally present in embryonic CMs. At the early stages, these structural changes exert a compensatory mechanism that maintain cardiac output (CO) and preserve RV function. However, these adaptive mechanisms no longer suffice and become overwhelmed by biomechanical stress, thereby resulting in enhanced oxidative stress, inflammation, and fibrosis, which culminates in electrophysiological changes/contractile dysfunction, loss of CMs and RV failure. (**B**) Simplified scheme of key factors initially engaged in the execution of CM hypertrophic response. Persistent and excessive stress impacts this initially adaptive core program (post-transcriptional modifications, isoform switching, distinct transcription factor combinations...) leading to its bifurcation to a maladaptive mode (failing CM). (**C**) Proposed model in which reactivation of fetal genes exerts either salutary or detrimental effects in function of their expression levels and stress magnitude.

Pulmonary hypertension (PH) represents a heterogeneous group of clinical entities defined as a mean pulmonary artery (PA) pressure above 20 mmHg and subcategorized into five groups by the World Health Organization [1]. Pulmonary arterial hypertension (PAH), which belongs to group 1 PH, is further subdivided into categories depending on the underlying etiologies; idiopathic PAH, heritable PAH, PAH related to various conditions, such as HIV infection, exposure to certain drugs and toxins, connective tissue disease, portal hypertension and congenital heart disease, as well as persistent PH of the newborn [1]. Although PAH can result from various triggers, all forms of PAH are characterized by sustained vasoconstriction and vascular remodeling of small PAs driven by exaggerated proliferation and resistance to apoptosis of resident cells (i.e., endothelial cells, smooth muscle cells and fibroblasts) [2]. The progressive narrowing of the vascular lumen elevates the PA pressure and consequently imposes a hemodynamic load on the right ventricle (RV). In the face of increased afterload, PAH patients initially develop concentric RV hypertrophy with preserved function; a mechanism called “adaptive or compensatory RV hypertrophy” [3]. Due to the inescapable rise in the pulmonary vascular resistance, this compensatory phase transitions to eccentric hypertrophy and chamber dilatation associated with cardiomyocyte cell death and pronounced fibrosis [3,4] (Figure 1). This process, referred to as “maladaptive RV hypertrophy or RV decompensation,” is associated with a progressive decline in cardiac function and death. Currently approved PAH therapies are primarily dedicated to combat pulmonary vasoconstriction and offer a limited survival benefit [5]. Thus, the development of therapeutic strategies aiming at blocking or reversing pulmonary vascular remodeling along with improving RV function is a pressing need that can only be achieved with a better understanding of the complex molecular mechanisms dictating pathological remodeling.

In the last decade, PAH has increasingly emerged as a disorder sharing numerous hallmarks of cancer [2,6]. As observed in cancer cells, excessive proliferation and survival of PA endothelial cells (PAECs) and mural cells, especially PA smooth muscle cells (PASMCs), driven by epigenetic reprogramming and fueled by a metabolic shift towards glycolysis and persistent inflammation [2,7,8], are key features involved in the obliteration of distal PAs in PAH. Interestingly, accumulating evidence suggests that these pathological conditions recapitulate the gene expression pattern found in the early developmental stages of the corresponding organ [9,10,11]. This is best exemplified by studies conducted in cancer cells demonstrating a reactivation of silent embryonic/fetal genes, normally repressed postnatally, promoting rapid growth of tissues for their own perverse purposes [12,13,14]. It is thus tempting to speculate by circular reasoning that pathological narrowing and obliteration of PAs inappropriately reprises feature of early lung development. This is also true for RV hypertrophy in PAH, which is intimately associated with suppression of the postnatal gene program and the concomitant up-regulation of a set of genes operating during cardiac development. Whether this genetic response exerts adaptive or detrimental functions is still an open fundamental question. In this review, we emphasize the potential contribution of genes and pathways normally involved during development in the pathogenesis of PAH. 

## 2. Reactivation of the Fetal Gene Program Is a Hallmark of Ventricular Remodeling

Cardiac hypertrophy is a stereotypic response of cardiomyocytes to increased workload which can be classified as physiological when due to hemodynamic demands on the neonatal heart, pregnancy or strenuous exercise training or as pathological when elicited by sustained pathological signals (e.g., hemodynamic overload) [15,16]. This process reflects an effort to alleviate the elevation in wall stress, according to Laplace’s principle. Although characterized by a continuum of adaptation, cardiac remodeling in PAH is classically dichotomized into two hypertrophic stages; an adaptive phase during which the cardiac function is preserved, followed by a maladaptive phase (due to further increases in hemodynamic overload) during which intense cardiomyocyte cell death and fibrosis accompanied by pronounced inflammation occur setting the stage for major decline in cardiac function (Figure 1) [17,18]. It is important to point out that the mechanisms underlying pathological RV remodeling have historically received less attention than its counterpart of the left side and that substantial differences exist between the left ventricle (LV) and RV including embryological origin, chamber geometry, and response to therapies [19] that may contribute to differential response under pathological conditions and rendering any extrapolation uncertain. While there is widespread consensus on the fact that hypertrophy can be either beneficial or detrimental, the molecular mechanisms and pathways governing the process from adaptive to maladaptive hypertrophy remain elusive.

### 2.1. Adult Ventricular Hypertrophy and Failure: A Reversion to a Fetal Pattern of Energy Substrate Metabolism

A major hallmark of the stressed heart is a return to a fetal-like pattern of energy substrate metabolism [20]. During early cardiac development, immature and proliferating cardiomyocytes mainly use anaerobic glycolysis for energy. During the perinatal period, cardiomyocytes gradually lose their proliferative capacity and concomitantly initiate a hypertrophic growth to accommodate the increased workload. The transition from proliferative to mature cardiomyocytes is driven by a changing energy substrate and utilization with fatty acid oxidation as the preferential source of energy [21]; a switch directly associated with mitochondrial reorganization (increased number and size). One of the major regulators of this metabolic shift include the hypoxia-inducible factor 1-alpha (HIF1α) [22]. Highly expressed and stabilized under low-oxygen conditions, as those experienced by the intrauterine environment, HIF1α promotes and enhances the glycolytic program by activating glycolytic genes such as glucose transporters, lactate dehydrogenase A (LDHA), hexokinase 2 (HK2) and pyruvate kinase muscle isozyme M2 (PKM2) and by modulating expression and activity of peroxisome proliferator-activated receptors (PPARs), PPAR gamma coactivator 1 (PGC1α), and heart and neural crest derivatives expressed 1 (HAND1), leading to repression of lipid oxidation [23,24]. With the oxygen-rich postnatal environment, downregulation of HIF1α occurs contributing to enhanced fatty acid oxidation. Similar to that observed in the stressed LV, adaptive RV hypertrophy in PAH is marked by a decline in fatty acid oxidation and increased reliance on glucose, as evidenced by fluorodeoxyglucose positron emission tomography and computed tomography scans [25,26] (Figure 1). Upon progression of heart failure, the up-regulation of mitochondrial glucose oxidative activity becomes inefficient to compensate the decrease of fatty acid oxidation and ultimately leads to the incapacity of the heart to fulfill its pump function. From a molecular standpoint, increased activity of the transcription factors cMyc [27] and HIF1α, enhanced expression of glucose transporters (GLUT1 and GLUT4) [26] along with the downregulation of PPARs have been shown to collectively account for the increased glucose uptake, glucose oxidation and repressed FA oxidation [28].

### 2.2. Fetal Genes as Cardiac Stress Markers

Alongside the metabolic changes reported, the molecular response that characterizes pathological cardiac hypertrophy and heart failure is commonly associated with a reactivation of well-described fetal genes, including atrial and brain natriuretic peptides (ANP and BNP) as well as the sarcomeric β-myosin heavy chain (β-MHC), while α-myosin heavy chain (α-MHC) and sarco/endoplasmic reticulum Ca^2+^-ATPase (SERCA2a) are diminished [29]. The functional consequences of the reciprocal regulation of α-MHC and β-MHC during cardiac remodeling, not obligatory coupled at the level of individual cardiomyocytes [30], is subjected of debate between those considering that this qualitative change actively contributes to heart failure and those suggesting that it represents a compensatory mechanism allowing the cardiomyocyte to be more energy-efficient [31]. As expression levels of the circulating hormones ANP and BNP originate from cardiac cells, positively correlate with the deterioration of hemodynamics and progression of clinical symptoms and dwindle as cardiac function improves [32,33], they are intensively used as biomarkers of cardiac stress/dysfunction. Studies have shown that secreted ANP and BNP activate the second messenger 3′-5′-cyclic guanosine monophosphate (cGMP) and exert pleiotropic functions, including inhibition of the renin-angiotensin-aldosterone system, reduction of endothelin-1 secretion and vasodilation of the systemic and pulmonary vasculature [34]. Their cardio-protective effect is underscored by numerous experimental and clinical observations. Indeed, mice with targeted disruption of BNP (*Nppb*^-/-^ mice) display spontaneous development of cardiac fibrosis [35], whereas inactivation of ANP (*Nppa* gene) or natriuretic peptide receptor A (NPR-A, the principal receptor of ANP and BNP) have been documented to worsen hypoxia-induced cardiac hypertrophy in mice [36,37]. Interestingly, an exaggerated hypertrophic response and marked deterioration of cardiac function were observed in mice subjected to aortic constriction and harboring a cardiomyocyte-specific deletion of the ANP receptor, compared to similarly treated control mice [38], thus providing a compelling evidence for a local cardioprotective effect. In view of these data and beyond their use as diagnosis and prognostic biomarkers in PAH, the high levels of ANP and BNP detected following cardiac stress may represent an unsuccessful attempt to counteract the maladaptive response by restraining hypertrophy and fibrosis.

### 2.3. Critical Implication of IGF1R/PI3K/AKT/mTOR Signaling in Both Physiological and Pathological Hypertrophy

Several intracellular signal transduction circuitries have been described playing a role during the adaptive response to cardiac stress. Among them, the insulin growth factor 1 receptor (IGF1R)-phosphatidylinositol 3-kinase (PI3K)–protein kinase B (AKT)-mammalian target of rapamycin (mTOR) axis is considered as one of the most important signaling cascades governing adaptive cardiac hypertrophy. Upon ligand binding, IGF1R activates several downstream signaling pathways, the two most prominent being PI3K/AKT/mTOR and mitogen-activated protein kinase/extracellular signal-regulated kinase (MAPK/ERK). Activation of the RAS/RAF/MEK/ERK signaling is associated with cell proliferation, whereas activation of PI3K/AKT/mTOR is classically depicted as a signaling cascade favoring cell growth and survival. AKT-dependent activation of mTOR involves the phosphorylation of tuberous sclerosis complex 2 (TSC2) and proline rich AKT substrate of 40 kDa (PRAS40) thereby relieving their inhibitory function on mTOR kinase activity. In turn, mTOR regulates numerous functions essential for cell homeostasis and adaptation via two functionally distinct multiprotein complexes named mTOR complex 1 (mTORC1) and mTOR complex 2 (mTORC2) that have both shared and unique subunits. mTORC1 positively controls cell growth and proliferation by stimulating protein synthesis, ribosome biogenesis, glucose uptake, and glycolysis, whereas mTORC2 mainly promotes cell survival [39]. Accordingly, elevated levels of cardiac IGF1 were observed in athletes exhibiting physiological cardiac hypertrophy compared to control subjects [40]. Necessary for cardiomyocyte proliferation and embryonic ventricular wall morphogenesis [41,42], the importance of IGF1R signaling in stimulating and maintaining physiological hypertrophy is further supported by experiments using genetically modified mice. Indeed, physiological cardiac hypertrophy induced by swimming exercise (a model producing mild growth of the ventricle with no evidence of interstitial fibrosis and no elevation of ANP and BNP [43]) was impaired in cardiomyocyte-specific IGF1R knockout mice [44], in mice expressing a dominant negative form of the p110α isoform of PI3K (dnPI3K) specifically in the heart [45] or in AKT mutant animals [46]. Conversely, concentric cardiac hypertrophy with preserved or enhanced systolic function and lack of fibrosis was found in mice overexpressing IGF1R or a constitutively active form of PI3K and AKT in cardiomyocytes [47,48,49]. In agreement with this, inactivation of phosphatase and tensin homolog (PTEN), which counteracts PI3K activity, resulted in compensated cardiac hypertrophy without dilation [50]. Under stress condition induced by pressure overload, enhanced expression of IGF1R or p110α was also cardioprotective when compared to nontransgenic mice [47,51]. Conversely, Zhang and collaborators found that inactivation of mTOR targeted to cardiac myocytes during adulthood prevented transverse aortic constriction (TAC)–induced hypertrophy and shortly induced severe cardiac dilatation [52]. A similar phenotype was observed after cardiomyocyte-specific deletion of Raptor (an essential component of mTORC1 complex) in adult mice [53]. In keeping with this, pharmacological inhibition of mTOR signaling using Rapamycin was documented to regress both compensatory and decompensatory cardiac hypertrophy in mice subjected to aortic constriction [54], whereas mTORC2 disruption was accompanied by suppression of compensatory cardiac growth, marked apoptosis and decreased contractile performance in response to pressure overload [55,56], underlying the overall importance of the two mTOR signaling branches in the adaptation of mechanical injury. That said, published data also reported that deletion of the mTORC1 activator Rheb1 in cardiomyocytes exerts protective effects against TAC-induced hypertrophy by reducing cardiac cell apoptosis [57] and that cardiomyocyte overexpression of PRAS40 results in blunted hypertrophic growth and decreased perivascular fibrosis in TAC-challenged mice [58], suggesting that prolonged activation of the AKT/mTOR signaling may play a detrimental role in cardiac remodeling. In the setting of PAH, up-regulation of mTOR signaling was documented as a feature of remodeled PA [59,60] and RV [61]. As a consequence, the dual pharmacological mTORC1/mTORC2 inhibitor PP242 demonstrated favorable effects with reduced pulmonary PA remodeling and PA pressure along with diminished RV hypertrophy and fibrosis in the Sugen/hypoxia (Su/Hx) PH rat model. Whether these observations resulted from direct cardioprotective effect on the RV or reduced PA pressure and subsequent decreased RV afterload remains to be confirmed. Furthermore, Shi et al. found that inactivation of *Igf1r* targeted to adult cardiomyocytes attenuates the increase in RV mass induced by hypoxia or PAB and improves RV functional parameters [62]. Taken together, it can be assumed that short-term activation of the IGF1R/AKT signaling represents a compensatory event in optimizing cardiac function, whereas it may exert detrimental effects on the heart and circulation activated over a long term.

### 2.4. Calcineurin and NFAT as Key Transducers of the Hypertrophic Response

Calcineurin is a calcium and calmodulin-dependent serine–threonine protein phosphatase composed of catalytic and regulatory subunits. When stimulated by a sustained rise in intracellular calcium, calcineurin catalyzes the dephosphorylation, and nuclear translocation of cytoplasmic nuclear factor of activated T-cells (NFATs); an intrinsic requirement for their biological activity. Necessary for proper cardiac development [63], the involvement of calcineurin and its downstream target NFAT in cardiac remodeling was initially described by Molkentin and collaborators [64]. In their study, mice expressing a constitutive active form of the calcineurin catalytic subunit driven by the αMHC promoter were generated. Transgenic mice exhibited a dramatic increase in heart size with accumulation of collagen surrounding cardiomyocytes, and reactivation of fetal genes and were more prone to sudden death. This in vivo hypertrophic response was corrected by chronic treatment with the calcineurin inhibitor cyclosporin A (CSA). Similar findings were noted in constitutive active NFAT3/c4 mutant mice [64]. Interestingly, subsequent studies revealed that, contrary to NFAT4/c3 activation, targeted disruption of NFAT3/c4 does not compromise the ability of the myocardium to undergo calcineurin-mediated hypertrophic growth, indicating that NFAT4/c3 operates downstream of calcineurin in the heart [65]. Importantly, using a calcineurin-responsive NFAT reporter mice, Cn/NFAT signaling was reported to participate in pathological, but not physiological, hypertrophy [66]. Following these observations, different studies have been undertaken to test the therapeutic potential of classical calcineurin inhibitors (CSA and FK506) in animal models of pressure overload. Mixed results have been obtained. Indeed, Shimoyama et al. found that FK506 (tacrolimus) totally prevents LV hypertrophy and fibrosis without affecting cardiac function in a pressure-overload rat model of abdominal aortic constriction [67], whereas two other studies demonstrated that LV hypertrophy induced by hemodynamic overload was unaffected by calcineurin inhibitors [68,69]. These seemingly conflicting results may, in part, be ascribed to differences between species, the inhibitors employed, the degree of inhibition, as well as probable off-target effects. Activation of calcineurin was also reported in the RV of mice subjected to PA banding (PAB) [70] and administration of CSA was shown to reverse established PAH in monocrotaline (MCT)-challenged rats by mitigating pulmonary vascular remodeling and improving cardiac functions [71]. The direct implication of calcineurin/NFAT signaling during the remodeling process of the RV was recently investigated by Boehm and collaborators. Using PAB in mice, the authors found that FK506 therapy initiated one week after PAB (when compensatory hypertrophy was established) significantly improved RV structure and function [72]. Considering the aforementioned literature and data demonstrating that CSA blocks the salutary role of compensated LV hypertrophy in aortic banded mice resulting in an increased susceptibility to decompensation and heart failure [73], ventricular activation of the calcineurin/NFAT signaling is likely an adaptive response that ultimately becomes maladaptive.

### 2.5. Other Transcription Factors Regulating Heart Development and Adult Ventricular Wall Stress

Another important factor is the transcription factor (TF) GATA Binding Protein 4 (GATA4), an essential regulator of cardiac morphogenesis [74] whose transcriptional activity is regulated through physical interaction with other TFs, such as NFAT, myocyte enhancer factor 2 (MEF2), HAND2, and NK2 transcription factor related locus 5 (NKX2.5). Overexpressed in the heart of neonatal mice compared to adults and upregulated during cardiac hypertrophy [75], GATA4 was identified as a key mediator of hypertrophy [76,77]. Indeed, *Gata4* loss of function targeted to the heart strikingly compromised the ability of the myocardium to hypertrophy and compensate to pressure overload or following exercise stimulation [76]. Likewise, *Gata4* hypomorphic mutant mice subjected to TAC exhibited eccentric hypertrophy, increased fibrosis and apoptosis, a phenotype partially corrected by IGF1R overexpression [77]. Along with GATA4, MEF2, which comprises four members (MEF2a, -2b, -2c, and -2d) generated by alternative splicing, is considered as a core cardiogenic TF and master regulator of cardiac hypertrophy. All members are expressed during cardiac development, while MEF2a and MEF2b are the most abundantly expressed isoforms in the adult heart. In the developing heart, MEF2 DNA binding activity peaks in the late fetal and neonatal periods and declines to low levels in adulthood. It increases de novo in conditions of pressure/volume overload [78]. Consistently, MEF2 is a key regulator of cardiovascular development, as illustrated by looping defects and absence of the RV in *Mef2c* null mice [79] and the high susceptibility for sudden cardiac death in mice lacking *Mef2a* [80]. Cardiac overexpression of *Mef2a* and *Mef2c* also induced cardiac hypertrophy and dilation [81,82]. In these models, the severity of the phenotype was correlated with transgene expression. To gain insight into the relevance of MEF2c in the pathophysiology of LV failure, Pereira and colleagues examined the expression levels of splicing variants of MEF2. They found that full-length and transrepressor γ domain-containing MEF2c transcripts were augmented in samples of failing as compared to those of healthy human hearts, while those lacking the γ fragment were significantly decreased [83]. Subsequent experiments conducted in cardiomyocytes revealed that the overexpression of MEF2c(γ+), but not MEF2c(γ-), leads to extensive sarcomeric disassembly and apoptosis. Accordingly, the authors demonstrated that transgenic mice previously published and exhibiting dilated cardiomyopathy secondary to overexpression of Mef2c [81], expressed, in fact, the γ fragment, whereas mice overexpressing MEF2c(γ-) appeared phenotypically normal [81].

By closely monitoring RV function in the MCT rat model of PAH, Paulin and collaborators showed that MEF2c sharply increases during the compensatory phase, whereas it falls at the decompensated stage [84]. In this study, the upregulation of nuclear receptor corepressor 1 (NCOR1), a transcription regulator known to recruit histone deacetylases and established repressor of cardiac hypertrophy [85], was proposed to account for the diminution of MEF2c in decompensated RV. Based on the literature documenting that MEF2c regulates a large set of genes involved in muscle contraction and metabolism, it is reasonable to expect that enhanced MEF2c activity is necessary to maintain RV function and that its downregulation contributes to RV failure. Given that myocardial-specific deletion of *Mef2c* driven by the αMHC-Cre results in viable offspring with no overt phenotype [86], assessing RV adaptation in these mutant mice in the face of pulmonary-independent RV-pressure overload may provide valuable information regarding its function. In addition to its expression level, acetylation of MEF2c also plays a critical role in coordinating the proper response of cardiomyocytes to hypertrophic stimuli. This was highlighted in a study showing that MEF2c acetylation is elevated in human LV samples from failing hearts compared with their non-failing counterparts and that expression of an acetylation-defective mutant form of MEF2c forestalls cardiac myocyte hypertrophy in culture. The authors also demonstrated that blocking MEF2c acetylation attenuates hypertrophy, fibrosis and cell death in mice subjected to TAC [87]. Apart acetylation, MEF2 may undergo various post-translational modifications, such as phosphorylation, methylation, SUMOylation, and ubiquitination contributing to the precise regulation of its stability and activity [88], which gives further evidence that MEF2 response is complex and regulated at multiple levels. Other TFs involved in cardiac development and implicated in cardiac cell hypertrophy include HAND2 and NKX2.5. Essential for RV development [89], HAND2 protein was found to be quickly increased in RV hypertrophy due to PAB in adult rats and maintained elevated during approximately 2 weeks. A similar pattern was noted for GATA4, MEF2, and NKX2.5 [90], strengthening the notion that these TFs, through the integration of information from a variety of signals and combinatorial interactions, act as lynchpins to cooperatively regulate a large number of genes (including fetal genes [91]) necessary for the adaptational response of the adult RV to pressure overload.

### 2.6. Epigenetic Regulation of the Fetal Gene Program

Regulation of gene expression requires the orchestrated effort of not only TFs, but also the dynamic interplay between chromatin-modifying enzymes (that add or remove epigenetic marks and referred as “writers” or “erasers”, respectively) and a class of proteins called “readers” that translate the epigenetic signal. While the role of a set of key TFs in cardiac remodeling has been actively investigated, the role played by epigenetic modifications, such as DNA methylation and histone modifications, is a relatively new field. Post-translational modifications of histones lead to changes in chromatin architecture with some of them promoting an open chromatin state called euchromatin allowing access of DNA for transcriptional machinery and subsequent gene activation and other exerting opposing effects. Up to now, acetylation and methylation of histone tails represent the most intensively studied post-translational modifications.

The acetylation state of histone is reversibly regulated by histone acetyltransferases and histone deacetylases (HDACs). P300 and its paralog CREB-binding proteins (CBP) are global transcriptional coactivators known to serve as scaffolds or molecular bridges between various TFs and the basal transcription machinery and exhibiting intrinsic acetyl transferase activity on nucleosomes and various factors. Several lines of evidence indicate that P300 plays a pivotal role in cardiac myocyte growth, starting with the fact that mice deficient for *p300* or harboring a single acetyltransferase-mutated allele display embryonic lethality due, at least in part, to heart defects [92,93]. Increased expression of P300 was found during the postnatal period of physiological hypertrophy, reaching a maximum at two months and declining thereafter. Upregulation of P300 was also detected in cardiomyocytes stimulated by norepinephrine, mice subjected to TAC and LV tissue from patients with end-stage cardiomyopathy [94]. To investigate the significance of this increase in the modulation of cardiac growth, distinct transgenic lines overexpressing different amounts of P300 were generated. These different mouse lines exhibited a dosage-dependent phenotype characterized by hypertrophy and heart failure. In mice overexpressing P300 at moderate levels, cardiac hypertrophy was well tolerated with lack of SERCA2 diminution and fibrosis. Despite this, most of these mice develop heart failure within a year. In mice expressing higher levels of P300, death occurred sooner. By contrast, haploinsufficiency for P300 was shown to limit TAC-induced hypertrophy [94]. This suggests that, although P300 drives adaptive hypertrophy, prolonged and/or increase in P300 levels above a certain threshold promotes decompensation. At the molecular level, P300-mediated acetylation of histones was shown to play an important role in the regulation of GATA4 expression in cardiogenesis [95]. Additionally, P300 was reported to acetylate several key TFs, including GATA4 and MEF2C, enhancing their transcriptional activity and *per se* the upregulation of fetal genes [94,96,97].

The histone methyltransferase enhancer of zeste homolog 2 (EZH2), being part of the polycomb repressive complex 2, is known to catalyze the trimethylation of lysine 27 of histone H3, resulting in gene silencing by chromatin compaction. While the molecular function of EZH2 during cardiac development is far from well-understood, studies have revealed that mice in which EZH2 was inactivated in the anterior heart field mesoderm (which contributes to the RV) develop progressive RV enlargement and fibrosis after birth. Mechanistic studies demonstrated that EZH2 directly represses the transcription factor Six1, an inducer of cell hypertrophy and activator of fetal cardiac genes. Using a genetic approach, the authors demonstrated that reduced Six1 dosage significantly rescues postnatal heart defects in Ezh2 mutant mice [98], highlighting the importance of SIX1 in pathological RV remodeling. Consistent with this, overexpression of Six1 led to adverse cardiac remodeling, whereas its knockdown attenuated pressure overload-induced cardiac dysfunction [99]. The critical role of EZH2 in the prevention of cardiac remodeling and dysfunction is further illustrated by a recent observation showing that (i) EZH2 presents a biphasic expression pattern during the natural course of RV remodeling in the setting of PAH, being upregulated in the human and rat compensated RV, and then downregulated in decompensated PAH RV; and (ii) the cardioprotective effects elicited by knockdown of the long non-coding RNA H19 in two animal models of RV failure is accompanied by an up-regulation of EZH2 [100]. Altogether, these data suggest that the reawakening of EZH2 is initially protector limiting maladaptive RV remodeling in PAH and that its subsequent downregulation hastens RV failure. In direct connection with this, H19, known as a fetal gene, was found to be upregulated in the decompensated RV from PAH patients and to precipitate RV failure. Moreover, circulating H19 levels in plasma was shown to discriminate PAH patients from controls, correlate with RV function, and predict long-term survival in two independent idiopathic PAH cohorts [100]. Surprisingly, H19 expression profile and outcome of its inhibition are totally opposite in the context of overloaded LV versus RV [100,101], thus highlighting how is uncertain to systematically extrapolate findings from one side to the other.

Reactivation of fetal cardiac genes programs in the hypertrophied adult heart also affects the expression of miRNAs. miR-208a and miR-208b are respectively encoded within α- and β-MHC genes. As the expression of miR-208a and miR-208b parallels the expression of their respective host genes [102], it is not surprising that the late phase of RV decompensation was associated with decreased miR-208a expression and concomitant up-regulation miR-208b expression [84]. In this study, increased MEF2c expression during the compensated phase was proposed to account for the subsequent diminution of miR-208a as part of the activation of the fetal gene program (i.e., switch from α to β-MHC). In turn, reduced miR-208a levels were suggested to induce the expression of the Mediator complex subunit 13/Nuclear receptor Corepressor 1 axis leading to repression of MEF2c and the entrance into a decompensated phase [84]. Although somewhat inconsistent with this study, inactivation of miR-208a in mice was found to be protective against TAC-induced LV hypertrophy and fibrosis [103] and silencing of miR-208a expression using antisense oligonucleotides during hypertension induced-heart failure in Dahl hypertensive rats was accompanied by diminished expression of β-MHC, reduced cardiomyocyte hypertrophy and fibrosis as well as improved cardiac function and survival [104]. Regarding miR-208b, the latter was identified as a pro-survival factor counteracting hypoxia-induced CM apoptosis [105], suggesting that its progressive increase during the course of RV remodeling is part of a protective mechanism.

## 3. Pulmonary Vascular Remodeling in PH: A Reawakening of Developmental Pathways?

During the last 10 years, a resemblance in the molecular mechanisms underlying development of hyperproliferative diseases and organ formation has surfaced, leading to the hypothesis that pulmonary vascular remodeling could be driven by inappropriate activation of a set of conventional signaling pathways used to build the lungs during morphogenesis.

### 3.1. Implication of Canonical Wnt Signaling

The Wingless and INT-1 (Wnt) signaling pathway is an evolutionary conserved system regulating multiple aspects of tissue development and homeostasis. Wnt signaling has been broadly separated into two branches: the β-catenin-dependent (canonical) and the β-catenin-independent (non-canonical) pathways [106]. Canonical Wnt signaling involves complex intracellular events culminating in cytoplasmic stabilization of β-catenin, which then translocates to the nucleus where it complexes with transcription factors and coactivators to initiate the transcription of target genes [106]. During lung morphogenesis, the canonical Wnt signaling pathway has been shown to be required for the differentiation of vascular smooth muscle cells. Indeed, mice conditionally deleted for β-catenin in smooth muscle precursors displayed a thinner smooth muscle layer surrounding the developing blood vessels [107]. This anomaly was accompanied by a reduced expression of Tenascin C (TnC), an extracellular matrix molecule stimulating platelet-derived growth factor receptor beta (PDGFRβ, a marker of smooth muscle precursors) expression. In agreement with these findings, increased expression of the Wnt/TnC/Pdgfr pathway was found in human PAH [107,108,109]. Similarly, epithelial deletion of *Grp177* was found to affect pulmonary vasculature development and reduce the proliferation of mesenchymal cells [110]. Indeed, *Grp177* mutant mice exhibited severe pulmonary hemorrhage as a result of reduced number of smooth muscle positive cells surrounding blood vessels associated with reduced expression of TnC. The implication of TnC is further stressed by studies showing that mutation of BMPR2 (the main genetic cause of familial PAH) induces TnC expression [109].

### 3.2. Pivotal Role of NOTCH Signaling in Lung Development and Homeostasis

The Notch signaling, consisting of four receptors (Notch 1-4) and five canonical ligands, is a conserved pathway that regulates cell-fate determination and proliferation. Notably, the Notch signaling was documented to be essential in mediating vascular endothelial cell to smooth muscle cell communication and thus development and maintenance of the vasculature [111]. Because of the importance of Notch signaling in vascular smooth muscle cell homeostasis, it is not surprising that it has gained much attention in the context of PAH. Indeed, NOTCH3 and its downstream effector Hairy and enhancer of split 5 (HES5) were found to be overexpressed in PASMCs from PAH patients and mice exhibiting *Notch3* loss-of-function mutation do not develop PH in response to chronic hypoxia [112]. In support of this, forced expression of a constitutively active NOTCH3 in normal PASMCs was shown to significantly stimulate their proliferation [112,113], whereas pharmacological inhibition of the signaling pathways was accompanied by a phenotypic conversion from a synthetic to a contractile phenotype [114]. In addition to NOTCH3, expression of its homolog NOTCH1 was found to be increased in endothelial cells from PAH patients compared to controls promoting the growth of PAECs while concurrently inhibiting their apoptosis [115]. Consistently, inhibition of the Notch signaling using a soluble Jagged 1 (that acts as a competitive inhibitor of Notch signaling) or γ-secretase (that blocks the proteolytic activation of NOTCH receptors) inhibited proliferation of PA cells and attenuated the development of PAH in multiple models [115,116].

### 3.3. Similarities in Transcriptional and Epigenetic Mechanisms Underlying Lung Development and Pulmonary Vascular Remodeling in PAH

#### 3.3.1. Hypoxia-Inducible Factors (HIFs)

Given that in utero lung development occurs in a low oxygen (relative hypoxic) environment, it is not surprising that HIF-1α and HIF-2 α play an important role in determining the organ shape. Studies conducted in genetically-modified mice along with early embryonic lung organ culture experiments have revealed that HIFs promote cell proliferation and cell survival and, by doing so, stimulate branching morphogenesis, vascularization and maturation [117,118]. Accordingly, increased expression of GLUT1/4, two transcriptional targets of HIFs, were found to be increased during fetal lung development in comparison to normal adult lung tissues [119]. Overexpression of GLUTs and the resulting increased glucose uptake provide energy requirements necessary to support the high rate of cell division during early lung morphogenesis and the synthesis of pulmonary surfactant phospholipids later during gestation. A greater activation of HIF-1α and HIF-2α is also seen in hyperproliferative and apoptosis-resistant PAH cells favoring the transactivation of glycolytic genes and energy shift [120,121]. As such, suppression of HIFs signaling has repeatedly demonstrated to alleviate PAH in experimental models [122,123,124]. Nonetheless, although bolstered by strong preclinical data, the potential clinical value of HIF inhibition has never been tested in PAH patients.

#### 3.3.2. Forkhead Box Protein M1 (FOXM1)

**Figure 2:**
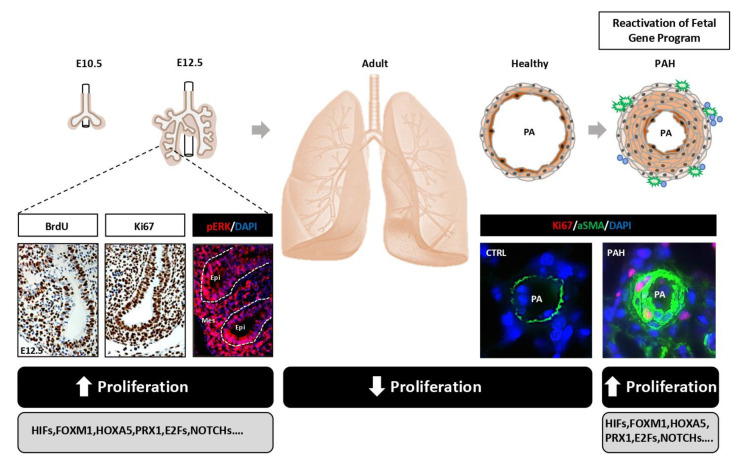
**Scheme illustrating the concept of reactivation of genes devoted to promote fetal lung growth in PAH.** A significant fraction of fetal genes fueling the explosive growth of the developing lung is reactivated/derepressed in PAH leading to pulmonary vascular remodeling and occlusion of distal pulmonary arteries.

FOXM1 belongs to a large family of transcription factors known as Forkhead transcription factors, widely expressed in actively proliferating tissues during embryogenesis [125] and overexpressed in many cancers [126]. To circumvent widespread organ defects and associated embryonic lethality of *Foxm1* null mice [127], a targeted loss of function approach was used to assess its role in the development of the smooth muscle layer during pulmonary vascular development. Indeed, the *Foxm1* gene was conditionally inactivated in mice using the Cre recombinase transgene driven by the smooth muscle myosin heavy chain (smMHC) promoter [128]. Mutant mice exhibited extensive pulmonary hemorrhage detected after transition of fetal to neonatal circulation (increase in the lung blood flow) compromising their viability. Although the differentiation of pulmonary smooth muscle cells was not affected by the loss of *Foxm1* function, reduced vascular smooth muscle cell proliferation and increased apoptosis was specifically detected indicating that FOXM1 is necessary for survival of differentiated pulmonary vascular smooth muscle cells. Based on these published results, its implication in PAH has been confirmed by several groups. Indeed, FOXM1 was found upregulated in both human and experimental PAH, promoting PASMC proliferation and resistance to apoptosis by stimulating the expression of cell cycle regulated proteins and DNA repair factors [129,130] (Figure 2). More importantly, both genetic and pharmacological inhibition of FOXM1 ameliorated pulmonary hemodynamics and histological changes in multiple animal models [129,130].

#### 3.3.3. Paired-Related Homeobox 1 (PRX1) and Homeobox A5 (HOXA5)

During lung morphogenesis, the paired-related homeobox gene PRX1 was also shown to promote pulmonary vascular smooth muscle cell differentiation by regulating the biochemical properties of the extracellular matrix [131]. Although not detected in normal adult rat PAs, de novo expression of PRX1 was reported to occur in the PA adventitia and within the media of remodeled PA from MCT-treated rats [132] and *Smad8* mutant mice [133]. Functional studies revealed that PRX1 stimulates PASMC proliferation and TnC expression contributing to the occlusion of the vascular lumen. Similarly, increased expression of the TF homeobox A5 (Hoxa5) was observed in human PAH tissue with strong immunoreactivity in concentric lesions [134]. Although the direct implication of HOXA5 in PAH remains to be established, its requirement in mesenchymal cell proliferation during lung morphogenesis [135] indicates that its upregulation in adults with PH may directly contribute to PA remodeling.

#### 3.3.4. Histone Deacetylases (HDACs) and Downstream Targets

The implication of epigenetic factors in the early and rapidly proliferative phases of lung development has been extensively studied. Among them, the histone deacetylases HDAC1 and 2 have been shown to be highly and broadly expressed at the pseudoglandular stage and contribute to the cell cycle progression by inhibiting retinoblastoma protein (Rb); a well-established transcriptional repressor of cell cycle genes containing E2F1 sites [136]. In PAH cells, up-regulation of HDAC1 [137] along with inactivation of Rb and increased expression of E2F transcription factor 1 (E2F1) target genes (i.e., CDK1, CCNA2…) have been shown to promote cell proliferation [138], reminiscent of their roles during lung development. Accordingly, genome-wide transcriptomic and proteomic profiling repeatedly showed an enrichment of genes related to the promotion of cell cycle progression, DNA replication and mitosis in PAH cells [139,140,141].

## 4. Concluding Remarks and Perspectives

To date, the molecular mechanisms of RV failure in PAH have received poor attention from the research community, with most efforts focused on the LV or directed towards understanding and reversing PA remodeling. Based on the literature, it can be assumed that both adaptive and maladaptive hypertrophy of the RV and LV are largely driven by a similar generic response characterized by the reactivation of the fetal gene program and concomitant suppression of the postnatal gene program, primary related to the structure and function of the cardiomyocyte [142]. Due to the prolonged and enhanced nature of the stress combined with circulating factors released from the diseased pulmonary vasculature, activation of a fibro-inflammatory maladaptive program occurs, which, grafted on the hypertrophic response, directly modifies the trajectory of cardiomyocyte remodeling, disrupts their integrity, and gradually promotes maladaptation [143]. The re-expression of fetal genes during cardiac remodeling is not limited to the aforementioned anecdotal examples (e.g., ANP, BNP, βMHC, GATA4, MEF2, P300…), but seems to be a generalized process. Indeed, by profiling histone marks that predict active enhancers using chromatin-immunoprecipitation sequencing followed by RNA sequencing on heart tissues from human fetuses, adult healthy patients and patients with dilated cardiomyopathy, hundreds of fetal genes, and more, a thousand fetal enhancers were shown to be reactivated in adult heart disease [144]. Along with transcriptional regulatory mechanisms, mRNA splicing patterns normally associated with heart development was shown to recur as part of the hypertrophic response to pressure overload [145]. It is presumed that this adult-to-fetal gene expression switch represents an initial salutary adaptation to stress, as most of these factors contribute to cardiomyocyte contractility, energy metabolism, growth and survival [142]. In line with this, it can be hypothesized that the reactivation of fetal genes is insufficient to counteract the maladaptive signals, or alternatively, once reach a certain level, turns on the dark side promoting maladaptive changes and organ dysfunction (Figure 1).

Significant gaps in our knowledge remain with respect to the molecular mechanisms which balance the adaptive and maladaptive responses of the RV. As getting compensated RV tissue samples from PAH patients is highly challenging, examination of temporal changes in gene expression with specimens hemodynamically characterized at the time of sacrifice (avoiding batch effects), and subsequently categorized into adaptive or maladaptive phase may provide a valuable tool to appreciate the role of the said factor throughout the remodeling process and, thus, prevent misinterpretation of the data. Indeed, examining RNA or protein levels as a snapshot at the time of sacrifice (most often at a decompensated stage) may indicate that a factor “X” is increased in the failing RV. However, a longitudinal analysis of the same factor can demonstrate that, although increased compared to healthy tissues, it is markedly decreased when compared to the compensated stage.

To reach an appropriate size, the fetal lung is the locus of massive cell proliferation. In this regard, it is thus not surprising that the reawakening of a significant fraction of genes mainly devoted to promote cell proliferation and thus fueling the explosive growth of the fetus likely constitutes a driving mechanism of lumen obliteration (Figure 2). Although experimental evidence has pinpointed commonalities between lung development and pulmonary vascular remodeling, these parallels result from single gene or signaling pathway studies. Systematic approaches could provide valuable insight to gauge the extent of the overlap in gene expression between lung development and the remodeled pulmonary vasculature in PAH. Likewise, a better understating of how epigenetics influences the reactivation of fetal genes/the suppression of adult program may pave the way for new therapeutic solutions. As genes implicated in cell proliferation and differentiation play crucial roles during development, knockout of many of them is associated with embryonic/perinatal lethality [146]. Tissue specific or conditional gene targeting may represent a powerful tool to bypass the early lethality associated with global inactivation and thus to assess the contribution of genes during late development and PAH. Furthermore, genes essential for embryo and PA cell survival and proliferation and non-essential for adult life represent promising drug targets to selectively kill cells in remodeled PA without affecting normal cells.

In summary, the striking similarity in terms of gene expression between development and pathological cardiopulmonary remodeling along with the close relationship between developmental insults and occurrence of PH in adulthood [147,148] suggest that lessons gained from developmental sciences may be particularly valuable to unravel how the disease develops, and reciprocally.

## Data Availability

No new data were created or analyzed in this study. Data sharing is not applicable to this article.
